# Surgical Treatment of Retro-Displacements of the Uterus

**Published:** 1916-03

**Authors:** James A. Hill

**Affiliations:** Houston, Texas


### Surgical Treatment of Retro-Displacements of the Uterus.

BY DR. JAMES A. HILL, HOUSTON, TEXAS.

In the discussion of a subject of this importance we had better take up a general review of the surgical anatomy of the more principal organs involved; while it may prove dry, it will be time well spent.

Normally the uterus is slightly anteverted, and always firmly fixed to the vagina at the cervix, while the fundus is freely movable; it lies between the bladder in front and the rectum and the cul-de-sac of Douglass, "usually filled with intestines," behind. The peritoneal attachments of the uterus are of great importance to the surgeon. The peritoneal investment merges on either side to form the broad ligaments, which are attached laterally to the sides of the pelvis, dividing the latter into two compartments. The anterior fold blends with the utero-vesical fold at the level of the internal os, while the posterior fold extends more deeply behind to form the pouch of Douglass.

Surgically the broad ligaments contain five important structures; the free upper surface forms a peritoneal investment for the Fallopian tubes; below these lie the round ligaments; at the base the uterine arteries and ureters. In the posterior fold of the broad ligament, lying between the Fallopian tube above and the round ligament below is a thickened peritoneal fold—the infundibulo-pelvic or suspensory ligament of the ovary. This ligament attaches the ovary to the lateral wall of the pelvis and contains the ovarian artery. Posteriorly is a small fold of peritoneum—the utero-sacral ligament.

So far as the correction of the retro-displacement of the uterus is concerned the round ligaments are the most important of the above named structures, and their importance merits further description. The ligaments are cord-like structures which leave the uterus at the cornua, just below and anterior to the Fallopian tubes and pass upward, outward and forward to the inguinal ring, thence through the inguinal canal to end in the subcutaneous tissue and skin of the labia majora. These ligaments are composed chiefly of fibrous tissue, with a quantity of unstriped muscle near the uterine end.

Probably no place in the field of surgery demands better judgment in the selection of the operation of choice than in that of the correction of retro-displaced uteri. There are so many points to be considered in this selection that he who attempts operation by

"rule of thumb," without careful thought as to its suitability for the case in hand often meets with disappointing results. In this selection there are two important factors concerned, namely, the etiology of the displacement and the pathological condition found at the time of operation. Let it be understood at the start that the round ligaments were never intended by nature for suspensory ligaments of the uterus, but merely for guy ropes. The indiscriminate use of the round ligaments alone for the purpose of suspension is not to be commended.

In long standing cases of retro-displacements, which are purely surgical, in a large per cent we find more or less extensive perineal tears, with relaxed pelvic floor. Obviously a suspensory operation without repairing the relaxed pelvic floor, which is probably the primary cause of the condition, would give little permanent benefit. The question of future pregnancy is an important one, as the fixation or suspension of the uterus to the abdominal wall during the child bearing period is never advisable. On the other hand, if for any reason the case is a sterile one, suspension is the operation of choice, and the results are generally satisfactory. In cases which the round ligaments are stretched into thin attenuated cords, they are of little use and some other operation is necessary. In such condition the operation of choice is that devised by Coffey, which consists of a plication of the broad ligaments. The ligaments are seized near their outer margin, and below the Fallopian tubes with clamps, folded upon themselves, and sutured. Theoretically the only objection I see to this operation is the subsequent kinking of the tubes, though no such results have occurred that we have record of.

In those cases where the round ligaments are strong and well formed the Baldy-Webster operation is the one of choice by a number of surgeons. This simple operation consists of pulling the uterine ends of the round ligaments through holes made in the broad ligaments, and suturing them together with silk upon the posterior surface of the uterus, well up toward the fundus so the uterus cannot prolapse backward over the attachments. The most serious indictment of this operation is that it makes use of the proximate ends of the round ligaments, which contain practically all of the smooth muscle, which hypertrophy and elongate most during pregnancy.

In cases suitable for the utilization of the round ligaments, my preference is an operation done (probably first) by Barrett of Chicago,—the principle from an anatomical standpoint being very much in its favor, and in my hands has proven very satisfactory to myself and the patient. This operation avoids the serious objection to both the Gillium and Baldy-Webster operations. This consists, usual abdominal incision, pulling the uterus up in place—secure the round ligaments about the junction of the middle and outer third (or at a point in your judgment will hold the uterus in proper place). Pass a piece of silk or catgut under same, leaving this as a guide. Then make a slight dissection separating the belly from the outer sheath of the abdominal muscle, pushing a pair of forceps in this dissection hugging the under surface of the muscle sheath to the internal ring (with an assistant making traction on this guide which marks the inner surface of the internal ring) plunge through the internal ring with the forceps, catch the guide string, i. e., around the round ligament, pull through and attach to the under surface of the muscle sheath, then closing the abdomen in the usual manner.

In presenting this paper it is not my intention to report anything new, but to try and elicit a discussion that will bring out the things conducive to the better welfare of our women in such a common condition that confronts us daily.

